# Tuberculosis and patient gender: An analysis and its implications in tuberculosis control

**DOI:** 10.4103/0970-2113.48897

**Published:** 2009

**Authors:** Sukhesh Rao

**Affiliations:** *Department of Tuberculosis and Respiratory Diseases, Yenepoya Medical College, Deralakatte, Mangalore - 575 018, Karnataka, India*

**Keywords:** Tuberculosis, gender, control, reproductive health

## Abstract

**Objective:**

To analyze the profile of pulmonary tuberculosis patients with respect to gender and its implications in tuberculosis control. Setting: DOTS center at a tertiary, teaching hospital in South India.

**Materials and Methods:**

A retrospective study was undertaken by screening medical records of 446 patients with pulmonary tuberculosis. Data studied included age, gender, and sputum smear status. Patients with comorbid conditions were excluded. No other data were considered.

**Results:**

The male to female ratio in patients of pulmonary tuberculosis was 2:1, which was also maintained when smear positive and smear negative were studied separately. The ratio of smear positive to smear negative patients was statistically significant at 4.4:1. A large proportion of patients (65–68%) were in the young and reproductive age group. Approximately, one-fifth patients were in the geriatric age group.

**Conclusion:**

The observation that two-thirds of all female smear-positive patients were found in the young and reproductive age group has strong implications in tuberculosis control strategies because of higher chances of mother to child transmission and higher probability of complications because of attendant antenatal and postnatal morbidity. Geriatric patients comprise another significant group because of higher chances of default, complications, inconvenience, and existence of other comorbid conditions.

## INTRODUCTION

Malignant pleural mesothelioma is one of the rare neoplasms of pleura. In virtually all countries, more male than female cases of tuberculosis are reported.^1^ In most low-income countries, the ratio of male to female cases of tuberculosis is approximately 2:1,^2^ attributable to biological characteristics and socioeconomic and cultural barriers to access healthcare.^3^ World Health Organization has also encouraged more studies on tuberculosis in relation to patient gender, to determine whether women with tuberculosis are more likely to be diagnosed, treated, or reported.^4^

Various studies have shown different statistical figures in the clinicoradiological profile of tuberculosis patients with relation to gender.^5^–^7^ Hence, this study was carried out to analyze the clinical and bacteriological profile of patients of tuberculosis in relation to gender.

## MATERIALS AND METHODS

Patients of pulmonary tuberculosis diagnosed and treated at DOTS center in our hospital were included in the study. A retrospective analysis of records of such patients was undertaken. Data regarding age, gender, and sputum smear status were studied. Patients with coexistent morbid conditions like diabetes and immunosuppression were not included. No other details were taken into account.

## RESULTS

Records of 446 patients formed the study group. Out of these, 308 (69%) were of males and 138 (31%) of females. The breakup of smear-positive and smear-negative patients were 364 (82%) and 82 (18%), respectively. Two hundred forty eight (68%) of smear positives were males and 116 (32%) were females. The corresponding numbers among smear negatives were 60 (73%) and 22 (27%), respectively [Table 1]. Age-wise distribution of patients is shown in Tables 2 and 3. It is clear that the young and reproductive age group constituted a significant percentage, 65% for males and 68% for females. Approximately, 20% of patients belonged to the geriatric age group.

**Table 1:** Distribution of cases with respect to gender

	Number of patients (%)	Number of smear positive patients (%)	Number of smear negative patients (%)
Males	308 (68)	248 (68)	60 (73)
Females	138 (31)	116 (32)	22 (27)
Total	446	364	82

**Table 2:** Distribution of patients with respect to age group

Age group (years)	Males (%)	Females (%)
15–30	82 (26)	52 (38)
30–45	120 (39)	42 (30)
45–60	52 (17)	26 (19)
>60	54 (18)	18 (13)
Total	308 (100)	138 (100)

P<0.05

**Table 3:** Distribution of smear positive cases with respect to age group

Age group (years)	Males (%)	Females (%)
15–30	62 (24)	46 (40)
30–45	94 (38)	32 (28)
45–60	46 (19)	22 (22)
>60	46 (19)	16 (13)
Total	248 (100)	116 (100)

P<0.05

## DISCUSSION

The results of this study show that the male to female ratio in patients of pulmonary tuberculosis is 2:1, which is in concurrence with other reports.^2^,^3^ But the proportion of sputum positive to sputum negative is 4.4:1, which though significant is not in concurrence with various other reports, which state that in tuberculosis control programs, such ratio should be 1: 1. Whether this reflects the increased prevalence, under treatment of smear negative cases, or better quality of sputum collection and analysis is difficult to predict because of the small group and retrospective nature of the study.

Another significant observation is that the male to female ratio among sputum positive cases remains at 2:1, implying women are equally motivable to produce adequate quality sputum and be diagnosed as sputum positive. A recent study^7^ found the opposite finding and they attributed it to inadequate sputum collection and social stigma/cultural barriers in females.

Age-wise distribution of patients also brought out some interesting observations. Almost 60–65% of patients were found to be in the young and reproductive age group, more so among sputum positives and females, leading to the inference that more sputum positive females were diagnosed in this group. This has strong implications in tuberculosis control strategies.

Females in the reproductive age group are (a) at a higher risk of transmitting infection to the child, at times attributed to nursing, (b) at a higher risk of complications because of attendant antenatal and postnatal morbidity, especially in developing countries, and (c) at a higher risk of default, due to various reasons like work at home, childcare, inconvenience to visit DOTS center, etc.^7^

Same was the findings among males too. In developing countries and countries with high prevalence, due to various socioeconomic reasons like males being sole breadwinners, higher chances of being employed in the unorganized sectors, lesser chances of awareness about diseases, probability of default is high. Anecdotal evidence also suggests that males in reproductive age group have found it inconvenient to attend DOTS centers because of their work timings. All these aspects should be looked into and appropriate strategies be developed.

Last but not the least, approximately one-fifth of patients belonged to the geriatric age group (in both sexes). This group generally has coexistent systemic diseases, which makes them prone to complications of the disease, spread of disease, and difficultly in treating because of side effects involved. Moreover, the inconveniences to visit DOTS centers because of ill health and necessity of an accompanying attendant may lead to noncompletion of treatment and higher chances of drug resistance.

In conclusion, the results of this study show that in patients of pulmonary tuberculosis, (a) male to female ratio was 2:1, (b) smear positive to smear negative ratio was 4.4:1, (c) large numbers of patients were in the young and reproductive age group, and (d) approximately one-fifth of patients belonged to the geriatric age group. These findings become highly relevant and very significant in planning different strategies in tuberculosis control programs, especially in countries with high prevalence and poor resources.
